# Feasibility of Conservative Management for Intraperitoneal Bladder Perforation: A Single-Institution Case Series

**DOI:** 10.3390/healthcare13131594

**Published:** 2025-07-03

**Authors:** Zorawar Singh, Ella Taubenfeld, Theodoros Karanikolas, Andrea Moyer, David Chan, Manish Vira, Justin Shinyu Han

**Affiliations:** Smith Institute for Urology, Northwell Health, Lake Success, NY 11042, USA; etaubenfeld@northwell.edu (E.T.); tkaranikol@northwell.edu (T.K.); amoyer@northwell.edu (A.M.); dchan@northwell.edu (D.C.); mvira@northwell.edu (M.V.)

**Keywords:** bladder perforation, Foley catheter, conservative management

## Abstract

**Introduction and Objectives**: Bladder injuries are broadly classified based on anatomical location into two main categories: extraperitoneal and intraperitoneal. Traditionally, clinicians manage most extraperitoneal bladder ruptures conservatively with catheter drainage, while intraperitoneal ruptures are surgically repaired. This study aims to evaluate the feasibility of conservative management of intraperitoneal bladder rupture in the largest series to date. **Methods:** A retrospective review was performed of patients treated for intraperitoneal bladder perforations at two large tertiary care centers from 2015 to 2023. The charts of 290 patients with intraperitoneal perforations were reviewed to compile a list of those who underwent initial conservative management of their rupture via Foley catheter, of which there were 16. Demographic data was collected as well as variables related to patient characteristics, computed tomography (CT) measured size of perforation, management, complications, and follow-up. Data were analyzed using descriptive statistics, and comparative analyses (*t*-test and Fisher’s exact test) were performed. **Results:** Our final analysis identified 16 patients with intraperitoneal bladder rupture treated with initial conservative management. Of these patients, 15 (94%) were successfully managed with Foley catheter placement. Four patients (25%) experienced complications after conservative management, which included long-term urinary incontinence/retention, urinary tract infection (UTI), and pelvic abscess. For patients successfully managed conservatively, the median duration of catheterization was 18 days (IQR 21.75). **Conclusions**: For patients with small intraperitoneal bladder ruptures, conservative management with prolonged Foley catheterization is a suitable and successful strategy. Future studies evaluating outcomes in larger cohorts of patients will help determine whether this strategy should be considered more frequently in select patient populations.

## 1. Introduction

Bladder injury typically occurs as a result of trauma in the setting of rapid-deceleration motor vehicle collisions and is often associated with pelvic fracture [1]. Other etiologies for bladder injury include penetrating trauma, iatrogenic surgical complications, and spontaneous rupture in patients with a history of neuropathic disease [1]. Bladder injuries are managed differently depending on the location of injury, with the major differentiator being whether the injury is in the intraperitoneal or extraperitoneal cavity [2]. There are certain indications for immediate intervention, but, for the most part, extraperitoneal bladder injuries are managed conservatively with catheterization [3,4]. In contrast, guidelines recommend that all patients with recognized intraperitoneal bladder injuries undergo immediate surgical repair due to the increased risk of urinary ascites leading to sepsis, azotemia, peritonitis, and ileus [3,4]. Previous studies have shown a significant decrease (59%) in mortality with immediate operative repair [5]. However, in the past 10 years, there have been a handful of case reports that have shown that intraperitoneal bladder injuries can be successfully managed with conservative management alone [6,7,8,9].

In this study, we sought to highlight our institution’s experience in managing intraperitoneal bladder injuries conservatively with catheterization alone. This is the largest case series to date, with the aim of demonstrating the feasibility of conservative management for intraperitoneal bladder injuries. We also attempted to highlight patient-specific and injury-specific factors that could be used to predict success from conservative management alone, as well as long-term urinary outcomes/complications from conservative management.

## 2. Materials and Methods

A retrospective review was performed of patients treated for intraperitoneal bladder perforations at a single health system, which encompasses 21 hospitals, including two large tertiary care centers, from 2015 to 2023. A total of 290 patients were identified by ICD-10 codes for bladder injury and were then evaluated to determine if they met the inclusion criteria for the study. Inclusion criteria included patients who had experienced an intraperitoneal bladder rupture and who underwent initial conservative management with catheterization. Any patients who had extraperitoneal injuries or had intraperitoneal perforations managed initially with surgical intervention were excluded from the study. A total of 16 patients met the inclusion criteria and were subsequently analyzed for demographic data, clinical characteristics, characteristics of the intraperitoneal rupture (e.g., inciting event, size of perforation on computed tomography scan), and any complications following conservative management. For intraperitoneal perforations that were diagnosed with CT cystogram, perforation size was measured independently by two collaborators and then averaged to estimate the size of the defect. When applicable, pre- and post-catheterization creatinine values were recorded and compared with a *t*-test to determine if pre-catheterization acute kidney injuries (AKI) were responsive to catheterization. Subgroup analysis was performed comparing patients who had complications from conservative management versus those who did not to help determine if there were any demographic or clinical characteristics that may predict complications. Data were analyzed using descriptive statistics, and comparative analyses were performed with a *t*-test and Fisher’s exact test.

## 3. Results

**Figure 1:**
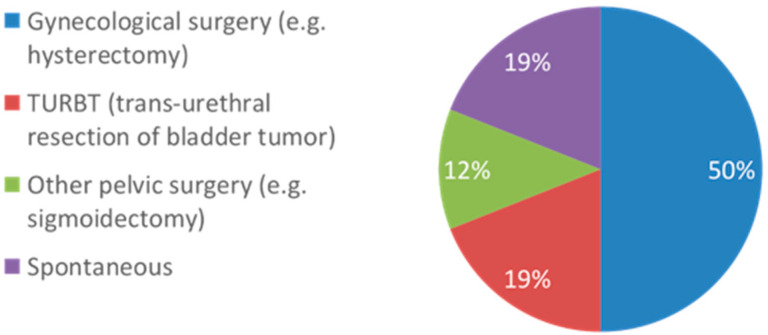
Incident events leading to bladder perforation among the final cohort of patients (n = 16).

A total of 16 patients with intraperitoneal bladder ruptures who underwent initial conservative management with catheterization were included in the final cohort. Baseline clinical and demographic data for the cohort are presented in Table 1. Etiologies leading to rupture included iatrogenic (*n* = 13.81%) and spontaneous (*n* = 3.19%). Procedures leading to iatrogenic rupture included hysterectomy, Cesarean section, sigmoidectomy, ileocecectomy, and transurethral resection of bladder tumor (Figure 1). All patients with gynecological procedures had injuries that were missed intra-operatively and then identified post-operatively. Diagnosis of intraperitoneal perforation was made via CT cystogram in 9 patients, with an additional diagnosis being made via direct visualization intra-operatively or via cystoscopy. For patients diagnosed via CT cystogram, the average size of conservatively managed perforations was 7.18 mm on computed topography (CT) CT scan (SD 5.18 mm). Patients presented with a variety of signs and symptoms, most commonly abdominal pain, acute kidney injury, and urinary retention. Time delay to presentation ranged from 0 to 29 days, with a mean of 8.5 days. Supplementary Figure S1 highlights the time to diagnosis from the suspected inciting event. After initial placement, the median duration of catheterization was 18 days (IQR 21.75). Catheterization also resulted in a statistically significant reduction in patients’ creatinine at presentation from a mean of 2.1 (SD 0.8) to 0.7 (SD 0.2), *p*-value < 0.01 (Table 2).

Three patients had simultaneous drain placement for management of abdominal fluid collection. A total of 15 patients (94%) had successful closure of their ruptures with conservative management alone, as confirmed by cystogram. One patient failed conservative management and required subsequent open cystorrhaphy. The patient requiring subsequent cystorraphy initially had a transurethral resection of bladder tumor for bladder cancer and was discharged from the hospital on post-operative day (POD) 0 without a Foley catheter. The patient was represented to the hospital on POD 3 with abdominal pain and fevers, with CT cystogram at the time showing an intraperitoneal perforation at the bladder dome measuring 1.5 cm in the largest dimension with an accompanying collection just superior to the defect. The patient was initially offered conservative management with catheterization and antibiotics, as with these measures, the patient’s abdominal pain and fevers quickly resolved. Patient remained asymptomatic and had repeat CT cystograms 1 week and 2.5 weeks after initial catheter placement, which showed a persistent leak, at which time the decision was made for operative intervention with exploratory laparotomy and cystorraphy. Intra-operatively, some necrotic tissue at the injury was debrided, and the bladder was closed primarily in two layers with subsequent resolution of bladder leak on repeat CT cystogram on post-operative day 7.

**Figure 2:**
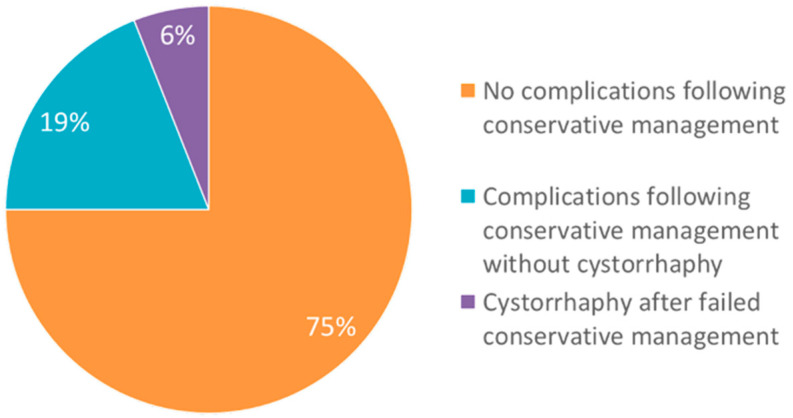
Outcomes of patients following conservative management of intraperitoneal bladder perforations expressed as a percentage of patients with and without post-treatment complications.

Patients were evaluated for any complications, which included infection/sepsis, additional procedural intervention, bleeding requiring transfusion, mortality, wound dehiscence, electrolyte disturbances, AKI, intensive care unit stay, readmission within 30 days, incontinence, or urinary retention. Four patients (25%) experienced complications after conservative management, which included long-term urinary incontinence/retention, UTI, and/or pelvic abscess (Figure 2).

## 4. Discussion

Bladder rupture can be extraperitoneal or intraperitoneal, with intraperitoneal rupture being the most common. The location of injury informs clinical management, with extraperitoneal ruptures typically managed conservatively with prolonged Foley catheter drainage and intraperitoneal ruptures managed with immediate surgical repair. To our knowledge, only four case reports are present in the literature that describe successful conservative management of intraperitoneal bladder ruptures with prolonged catheterization, particularly for patients in favorable clinical states–mild symptoms, small perforation, minimal urine infiltrations, and no significant infection [7]. Intraperitoneal bladder rupture has been reported to be successfully managed conservatively in a variety of rupture etiologies, including iatrogenic and spontaneous ruptures [6,7,8,9]. In our case series of 16 patients with intraperitoneal bladder rupture treated with initial conservative management, 15 were successfully managed with Foley catheter placement, with a median duration of catheterization of 18 days (IQR 21.75), which aligns with previous case report recommendations of at least 14 days of catheterization [7,9]. A total of 12 patients were managed conservatively without complications, while 4 experienced one or more complications. These included urinary incontinence/retention, UTI, and/or pelvic abscess.

Only one of the patients in this group underwent operative repair of the bladder wall after failed conservative management. For the patient who required operative intervention, their initial CT cystogram revealed a relatively small defect measuring 1.5 cm, but it was also associated with a pelvic collection. Initially, the patient responded well as symptoms (fevers, abdominal pain) resolved with catheterization and antibiotics alone. However, follow-up CT cystograms demonstrated a persistent leak at 1- and 2.5-week follow-up, prompting eventual conversion to open repair of the bladder defect. Upon exploratory laparotomy, the patient was noted to have purulence just superior to the bladder, as well as necrotic tissue at the site of the defect. The presence of the pelvic collection may have prevented adequate tissue healing and thus may explain the failure in conservative management [10]. Thus, based on our experience with this case, we recommend that if conservative management is utilized for intraperitoneal bladder perforations that any concomitant pelvic collections should be drained to prevent the development of pelvic abscesses and subsequent delayed tissue healing.

There were no significant differences between patients who experienced complications vs. those who did not, possibly due to the relatively small sample size of our cohort. However, patients who experienced complications with conservative management were on average older, more likely male, with higher body mass indices (BMI), with larger bladder defect sizes, had less decrease in creatinine with catheterization, and/or with a history of abdominopelvic radiation. Mechanism of injury in the entire cohort included iatrogenic (81%) and idiopathic spontaneous (19%) rupture, of which both etiologies were successfully managed. Procedures leading to iatrogenic injury included hysterectomy, Cesarean section, sigmoidectomy, ileocecectomy, and transurethral resection of bladder tumor.

There are single case reports that have highlighted the feasibility of successful conservative management of intraperitoneal bladder ruptures, but this is the largest case series to date [11,12,13]. The successful closure of rupture in 15 (94%) of these patients demonstrates that conservative management is a feasible strategy that may be considered in select patient populations. This is certainly a paradigm shift, as even recent studies have shown that the vast majority of intraperitoneal perforations are treated with surgical bladder closure, with only 17% of patients being offered conservative management with catheter drainage [14]. This study highlights that patients with relatively small intraperitoneal perforations, AKIs that respond to catheter placement, and minimal abdominal symptoms can be successfully managed with catheterization alone.

This study is limited by its small size and retrospective nature, which introduces selection bias and limits the generalizability of this study. Furthermore, given the small sample size, the subgroup analysis comparing patients with and without complications is likely not adequately powered to make meaningful conclusions. Another limitation of this study is that the measurement used to identify the size of the rupture was a CT cystogram, which only gives an estimation rather than the exact size of the defect. However, given the lack of research on this topic, this study is a strong lead point for future studies to analyze catheterization as treatment in larger intraperitoneal bladder rupture patient cohorts.

This article is a revised and expanded version of an abstract with the same title, which was presented at the AUA Annual Meeting 2024 at the Henry B. González Convention Center in San Antonio, TX, USA, 3–6 May 2024 [15].

## 5. Conclusions

Although current guidelines recommend immediate surgical repair for treatment of all intraperitoneal bladder perforations, the evidence supporting this practice is limited. At present, only case reports have described the successful conservative management of intraperitoneal bladder ruptures with prolonged catheterization. This case series is the largest to date demonstrating the feasibility of conservative management in select cases of intraperitoneal bladder ruptures. We highlight several factors that may impact the risk of complications and are important to evaluate in future studies, including the size of the rupture, patient age, response of AKI to catheterization, and patient BMI. Our cohort demonstrated successful closure of rupture with conservative management in all but one patient, emphasizing the feasibility of this strategy. Additional future studies evaluating outcomes in larger cohorts of patients are essential in determining if conservative management of intraperitoneal bladder injury should be considered more frequently in select patient populations.

## Figures and Tables

**Table 1:** Baseline demographic and clinical characteristics.

N	16
Age (mean)	54.6
Sex (n)	
Male (%)	4 (25%)
Female (%)	12 (75%)
Race (n)	
White (%)	7 (44%)
Non-white (%)	9 (56%)
BMI (mean, SD)	26.03 (6.4)
History of Hypertension (%)	4 (25%)
History of Diabetes (%)	2 (17%)
Mechanism of Injury	
Iatrogenic (%)	13 (81%)
Non-iatrogenic (%)	3 (19%)
History of Abdominopelvic Radiation (%)	2 (13%)
Cr on Presentation (mean, SD)	2.3 (1.8)

**Table 2:** Comparative analysis of patients managed conservatively who experienced no complications during or after management vs. those who experienced complications.

	No Complications	Complications	*p*-Value
N	12 (75%)	4 (25%)	
Age (mean)	50.9	64.8	0.09
Sex (% male)	20%	50%	0.24
Race (n)			0.26
White	4 (33%)	3 (75%)	
Non-white	8 (66%)	1 (25%)	
BMI (mean)	24.8625	29.54	0.11
History of Hypertension	3 (25%)	1 (25%)	1
History of Diabetes	2 (17%)	0 (0%)	1
Mechanism of Injury			1
Iatrogenic	10 (83%)	3 (75%)	
Non-iatrogenic	2 (17%)	1 (25%)	
Mean Size of Defect, mm (mean, SD)	6.5 (5.0)	11.1 (2.8)	0.26
Cr on Presentation (mean, SD)	2.1 (0.8)	3.2 (3.1)	0.19
Time to Presentation, days (mean ± SD)	9.2 (11.6)	4.5 (2.1)	0.30
Change in Cr with Catheterization (mean, SD)	2.1 (0.8)	0.7 (0.2)	0.01
History of Abdominopelvic Radiation	0 (0%)	2 (50%)	0.05

## Data Availability

The raw data supporting the conclusions of this article will be made available by the authors on request.

## Supplementary Materials

The following supporting information can be downloaded at: https://www.mdpi.com/article/10.3390/healthcare13131594/s1, Figure S1: Days to Presentation from Inciting Event.
