# Supplementary figures and images for: Feasibility of Conservative Management for Intraperitoneal Bladder Perforation: A Single-Institution Case Series
Source: Healthcare (Basel). 2025 Jul 3;13(13):1594. doi: 10.3390/healthcare13131594 (PMC12250053; doi:10.3390/healthcare13131594)

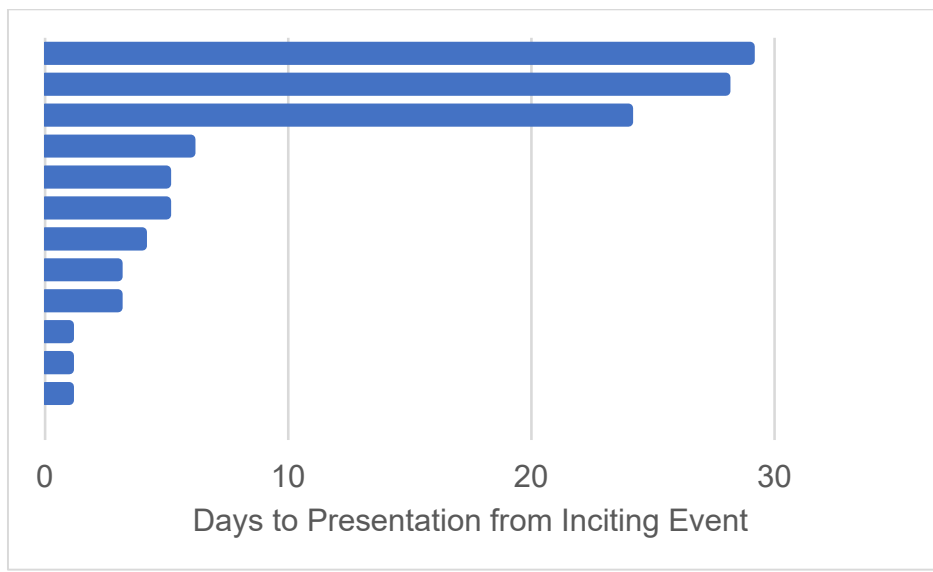

Supplementary Figure S1. Days to Presentation from Inciting Event

Supplement: Supplementary file 1 [file healthcare-13-01594-s001.zip › healthcare-3615341-supplementary.pdf]
